# Reduction of Selenite to Red Elemental Selenium by *Rhodopseudomonas palustris* Strain N

**DOI:** 10.1371/journal.pone.0095955

**Published:** 2014-04-23

**Authors:** Baozhen Li, Na Liu, Yongquan Li, Weixin Jing, Jinhua Fan, Dan Li, Longyan Zhang, Xiaofeng Zhang, Zhaoming Zhang, Lan Wang

**Affiliations:** 1 School of Life Science, Shanxi University, Taiyuan, China; 2 Shanxi Coal Geological Bureau, Taiyuan, China; RMIT University, Australia

## Abstract

The trace metal selenium is in demand for health supplements to human and animal nutrition. We studied the reduction of selenite (SeO_3_^−2^) to red elemental selenium by *Rhodopseudomonas palustris* strain N. This strain was cultured in a medium containing SeO_3_^−2^ and the particles obtained from cultures were analyzed using transmission electron microscopy (TEM), energy dispersive microanalysis (EDX) and X ray diffraction analysis (XRD). Our results showed the strain N could reduce SeO_3_^−2^ to red elemental selenium. The diameters of particles were 80–200 nm. The bacteria exhibited significant tolerance to SeO_3_^−2^ up to 8.0 m mol/L concentration with an EC_50_ value of 2.4 m mol/L. After 9 d of cultivation, the presence of SeO_3_^2−^ up to 1.0 m mol/L resulted in 99.9% reduction of selenite, whereas 82.0% (p<0.05), 31.7% (p<0.05) and 2.4% (p<0.05) reduction of SeO_3_^−2^ was observed at 2.0, 4.0 and 8.0 m mol/L SeO_3_^2−^ concentrations, respectively. This study indicated that red elemental selenium was synthesized by green technology using *Rhodopseudomonas palustris* strain N. This strain also indicated a high tolerance to SeO_3_^−2^. The finding of this work will contribute to the application of selenium to human health.

## Introduction

Selenium is a trace element commonly found in materials of the earth's crust, and it is essential for humans and animals. In human beings, the nutritional functions of selenium are achieved by 25 selenoproteins that have selenocysteine at their active center [1], [2]. In at least three prospective studies [3]–[5], high selenium status has been associated with a low overall mortality. Selenium supplementation, even in apparently selenium-replete individuals, has pronounced immunostimulant effects, including an enhancement of proliferation of activated T-cells, increased cytotoxic lymphocyte-mediated tumor cytotoxicity, and natural killer cell activity [6]–[11].

Selenium enters food chains typically by the absorbance of plants from soil. The daily selenium intakes of human mainly come from plants and animals containing selenium [12], [13]. But, selenium deficiency in soil is a worldwide problem. The distribution of a low selenium belt showed a zonal area in each hemisphere (latitudes above 30 degrees), especially in Europe and North America [14], [15]. Therefore, it is necessary to enrich the uptake of selenium for health reasons. But, there is a relatively narrow margin between selenium intakes that result in deficiency or toxicity [16]. A form of Se with higher bioavailability and lower toxicity may have better prospects as a chemo-preventive agent. Amongst its various forms, the red amorphous selenium is with biological activity and effects similar to those of sodium selenite but with much lower acute toxicity [15], [17]. It was considered the most potent chemical form to the artificial selenium enrichment. Therefore, the red amorphous selenium has attracted much attention [18]–[22].

In natural environments, live cells, such as bacteria, fungi, yeasts and plants, are known to be capable of converting selenate and selenite to Se^0^ [23]–[28]. Among these, bacteria are preferred for biosynthesis due to their extracellular particle production ability, short generation time, ease of culturing, downstream processing and manipulation [29]. *Rhodopseudomonas palustris* is a purple, non-sulphur, photosynthetic bacterium. It can grow not only aerobically in the dark but also anaerobically in the light [30] and has tolerance to a variety of metal and transition metal oxyanions, including selenium [31], [32]. This resistance is attributed to the capacity of the organisms to reduce Se oxyanions to their elemental ground state [29], [33], [34].

The objective of the present study was to investigate the ability of *Rhodopseudomonas palustris* strain N to reduce selenite to its elemental state. It will provide an eco-friendly and potentially economically viable ‘green’ synthesis route towards synthesis of red elemental selenium and contribute to the application of selenium for human health.

## Materials and Methods

### Ethics statement

The collection of sludge was permitted by Taiyuan sewage plant, Shanxi Province, China.

### Growth of bacteria

*Rhodopseudomonas palustris* strain N was employed in these experiments. This strain was originally isolated from sludge in Taiyuan sewage plant (112°33′E 37°52′N) and synchronously stored in the China General Microbiological Culture Collection [35]. Before the experiment, the strain was cultured in 500 mL bottles of full medium described by Ormerod [36] with the exception that DL-malate was omitted and 1.8 g sodium acetate was added. Bottles were closed by a rubber plug and the gas phase was exchanged with Ar by 5 cycles consisting of 1 atm of vacuum followed by the addition of Ar to a pressure of 1.5 atm. The cultures were incubated at 30 °C in the presence of incandescent light (1500 Lux) at gentle stirring (80 r/min) with magnetic stirrer. When it reached the end of the exponential growth phase (about 72 h after cultured), the cells were used to inoculate new culture flasks. Three transfers were done before the cultures were used for experiments.

### Transformation of selenite (SeO_3_^2−^) to Se^0^

The microbial transformation of SeO_3_^2−^ was studied under anaerobic conditions as mentioned above. *Rhodopseudomonas palustris* strain N was cultured under the same condition above, except that medium was supplemented with 1.0 m mol/L sodium selenite and the strain was incubated at 30 °C in the presence of incandescent light (1500 Lux) for 8 days. The volume of culture used for inoculation was calculated so that the starting cell concentration corresponded to an absorbance at 680 nm of 0.21 with a 10-mm path length.

### Scanning electron microscopy (SEM)

The strains were fixed in 2.5% glutaraldehyde for 48 h, rinsed three times in phosphate buffer (0.2 mol/L), and dehydrated in an ethanol series (30%, 50%, 70%, 90%, and 100%). Ethanol was displaced by isoamyl acetate, and the cells were dried using an EMS 850 critical point drying apparatus. Next, the samples were mounted on microscope slides (approximately 2.5 cm×2.5 cm) and sputter-coated with gold to a thickness of approximately 20 nm. Finally, they were observed under a JSM-6360LV scanning electron microscope (JEOL, Tokyo, Japan) operating at 30 kV.

### Transmission electron microscopy (TEM) and energy dispersive microanalysis (EDX)

The harvested cells cultured in the medium containing selenite were centrifuged at 12,000×g for 10 min. The precipitated pellet was collected and washed with normal saline. Cells were resuspended in 2.5% glutaraldehyde and fixed for 2 h, washed with phosphate buffer (0.2 m mol/L, pH 7.0), and embedded in low-melting-point agarose. Agar blocks (approximately 1 by 1 by 1 mm) were fixed in 1% OsO_4_ in running water for 60 min, dehydrated with ethanol and acetone, and embedded in Epon-Araldite. Sections cut from the Epon-Araldite preparation were contrasted with uranyl acetate and lead citrate as described by Hess [37]. For energy-dispersive X-ray (EDX) analysis, whole cells were applied to scanning electron microscopy grids, dried at room temperature, and coated with 5 nm of carbon before measurements were obtained. The samples were examined using a JEM-1011 scanning electron microscope (JEOL, Tokyo, Japan) with an accelerating voltage of 80 kV. The EDX analysis was performed with a JEM-2010 transmission electron microscope (JEOL, Tokyo, Japan) equipped with a Link-Inca microanalysis system.

### X ray diffraction analysis

The bacterial culture was centrifuged at 3,000×g and 4°C for 20 min after the reduction phase was completed. The pellet was discarded, and the supernatant containing Se^0^ in the form of Se-NPs was centrifuged at 100,000×g and 4°C for 30 min. The supernatant was discarded, and the pellet with the selenium-containing particles was resuspended in 50 m mol/L Tris-HCl (pH 7.5). The suspension was washed twice in the same buffer by repeating the two centrifugation steps. The pellet was freeze-dried on Thermo Dryer (Thermo, Savant ModulyoD-2300, USA) and stored in lyophilized powdered form until used for further characterization. The finely powdered sample was analyzed by a D8 Advance (Bruker, Germany) X-ray diffractometer using CuK_α_ radiation (λ = 1.5406 A) in the range of 5°≤2θ≤80° at 40 kV. In order to calculate the particle size (D) of the sample, the Scherrer's relationship (D = 0.9 λ/βcosθ) has been used [38], where λ is the wavelength of X-ray, β is the broadening of the diffraction line measured half of its maximum intensity in radians and θ is the Bragg's diffraction angle. The particle size of the sample was estimated from the line width of the (101) XRD peak.

### Resistance to SeO_3_^2−^ Stress

#### Bacteria culture

The effect of SeO_3_^2−^ on bacterial growth was determined by culturing the cells (the starting cell concentration corresponded to an absorbance at 680 nm of 0.21 with a 10-mm path length) in 500 mL bottle containing 500 mL of medium modification of described by Ormerod [36] supplemented with 1.0, 2.0, 4.0, and 8.0 m mol/L of Na_2_SeO_3_. The bottles were incubated at 30°C in the presence of incandescent light (1500 Lux). The growth in each bottle was sampled by at 0–5 days after cultured. The fermentation was used for bacterial biomass determination (calculated by cell concentration and protein content according to Kessi et al. [31]).

#### Protein content determination

The protein content of cells was determined by using a modification of the method of Lowry et al. [39]. 1 mL cultures were disrupted by ultrasonic treatment with 160 W for 15 min, at 4°C. A cell suspension of 200 µL was centrifuged at 15,000×g for 15 min, at 4°C and resuspended in 125 µL NaOH (0.1 mol/L). The resulting sample was frozen and stored. The samples were incubated in boiling water for 10 min. Then 875 µL of a solution containing 0.025% copper sulfate, 0.050% sodium tartrate, and 2.5% sodium carbonate was added, and the sample was incubated at room temperature for 10 min. After the addition of 250 µL of Folin-Ciocalteu solution (diluted 1/6 with H_2_O) and incubation for an additional 3 h, the absorbance at 750 nm of the copper-protein complex was determined by using a blank containing all of the reagents except protein. Selenium-containing samples were centrifuged at 15,000×g for 10 min before measurements were obtained. The optical density value was recorded using a microplate reader (M5, Spectra Max, www.spectramax.com). All measurements were done in triplicate. Bovine serum albumin was used as a standard. Growth ratio was calculated with a growth curve and the medium exposure concentration (EC_50_) [40], [41] was calculated using the Probit Analysis of Regression (Origin 8.0).

### Reduction of SeO_3_^2−^

Bacteria were cultured as described in ‘*bacteria culture*’ for 9 days. The fermentation product was centrifuged at 15,000×g for 15 min, at 4°C. The concentration of selenium in the supernatant fraction was determined following by the method reported by Zhang et al. [42]. The contents of the obtained supernatant was estimated by using LH-2A hydride generation (General research institute for nonferrous metals, Beijing, China) and AA-6300 atomic absorption spectrometer (Shimadzu, Tokyo, Japan) in an air-acetylene flame at 196.1 nm wavelength. Reagent channel: 2.0% KBH_4_/0.5% NaOH; Acid channel: 10% HCl; Speed of supported gas (N_2_): 200 mL/min. The samples were determined three times.

### Reduction of SeO_3_^2−^ in a cell-free spent medium

A 250 µL portion of 0.1 mol/L SeO_3_^2−^ was added to 25 mL of cell-free medium obtained from a 1.0 m mol/L SeO_3_^2−^ culture in which reduction was complete and culture without SeO_3_^2−^. The solution was thoroughly mixed and incubated at 30°C in the presence of incandescent light (1500 Lux) and observed after 120 h cultured.

### Statistical analysis

All data presented are the mean values of three independent sets of experiments. Each value was presented as means ± standard deviation (S.D.). Statistical analysis was carried out by one-way ANOVA using the Tukey's posthoc test to evaluate whether the means were significantly different. The level of significance was set at *P*<0.05. Statistical computations were performed with SPSS 16.0 for Windows (SPSS Inc.).

## Results

### Transformation of SeO_3_^2−^ to Se^0^

**Figure 1:**
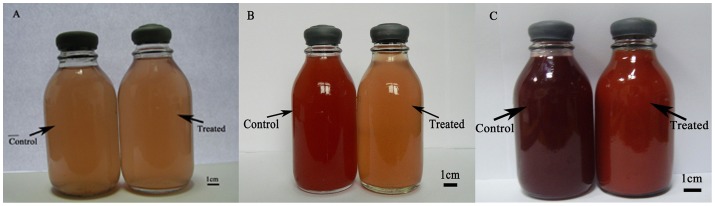
Culture of *Rhodopseudomonas palustris* strain N in medium containing selenite. A, at the start of cultivation; B, at the 2^nd^ day of cultivation; C, at the 8^th^ day of cultivation. Control: strain cultured in a medium without selenite; Treatment: strain cultured in medium containing selenite.

**Figure 2:**
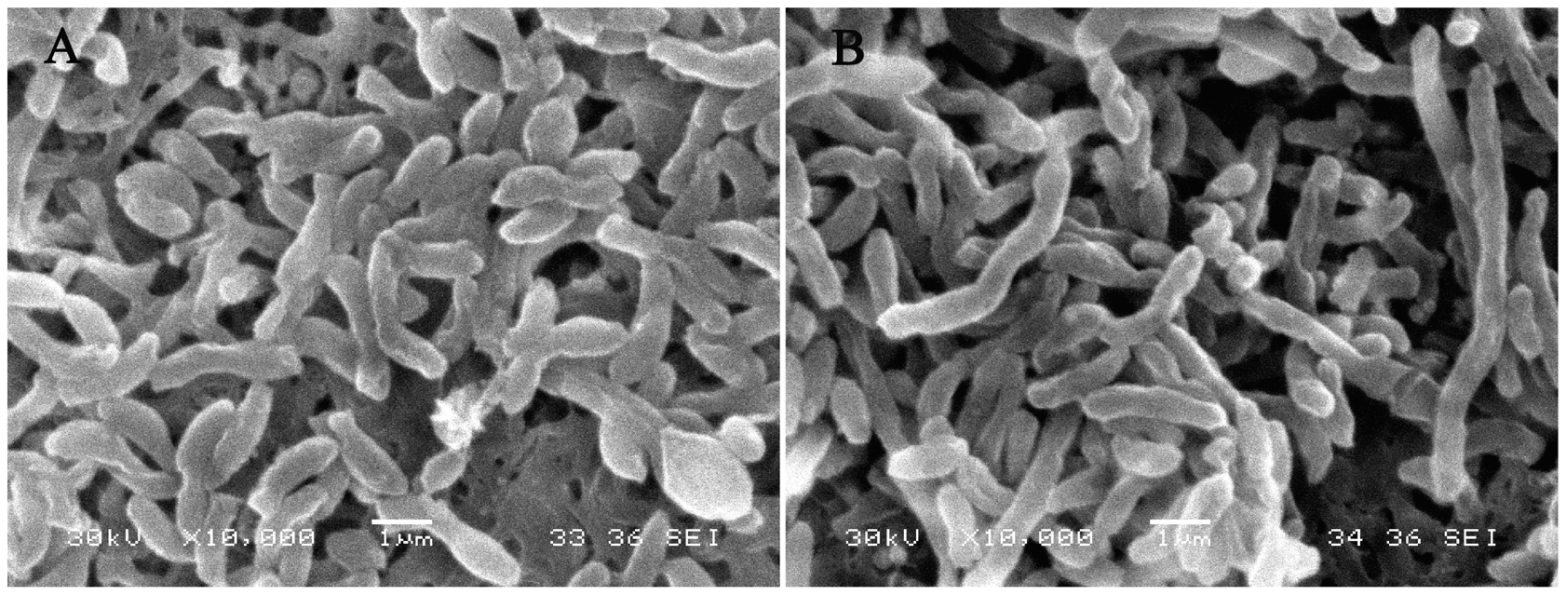
Cell morphology of *Rhodopseudomonas palustris* strain N cultured without selenite (A) and with 2.0 m mol/L selenite (B) under scanning electron microscopy.

*Rhodopseudomonas palustris* strain N was cultured in medium containing SeO_3_^2−^ under optimized growth conditions. At the start of culture, the culture with and without SeO_3_^2−^ had the same color (Fig. 1A). In the control group, the strain began to proliferation at the 1^st^ day after inoculated and shown a red color at the 2^nd^ day. But in the treated group, it began to grow and showed an orange color at the 2^nd^ day (Fig. 1B). At the 8^th^ day, the control group turned dark reddish brown (this color was mainly due to strain in the presence of chlorophyll a and carotenoids [43]) and the treated group exhibited a red color (Fig. 1C). Moreover, cells of strain N exhibited elongation when exposed to 2.0 m mol/L SeO_3_^2−^ (Fig. 2B) contrasting to the cells in the control group under scanning electron microscope (Fig. 2A).

### TEM and EDX analysis

**Figure 3:**
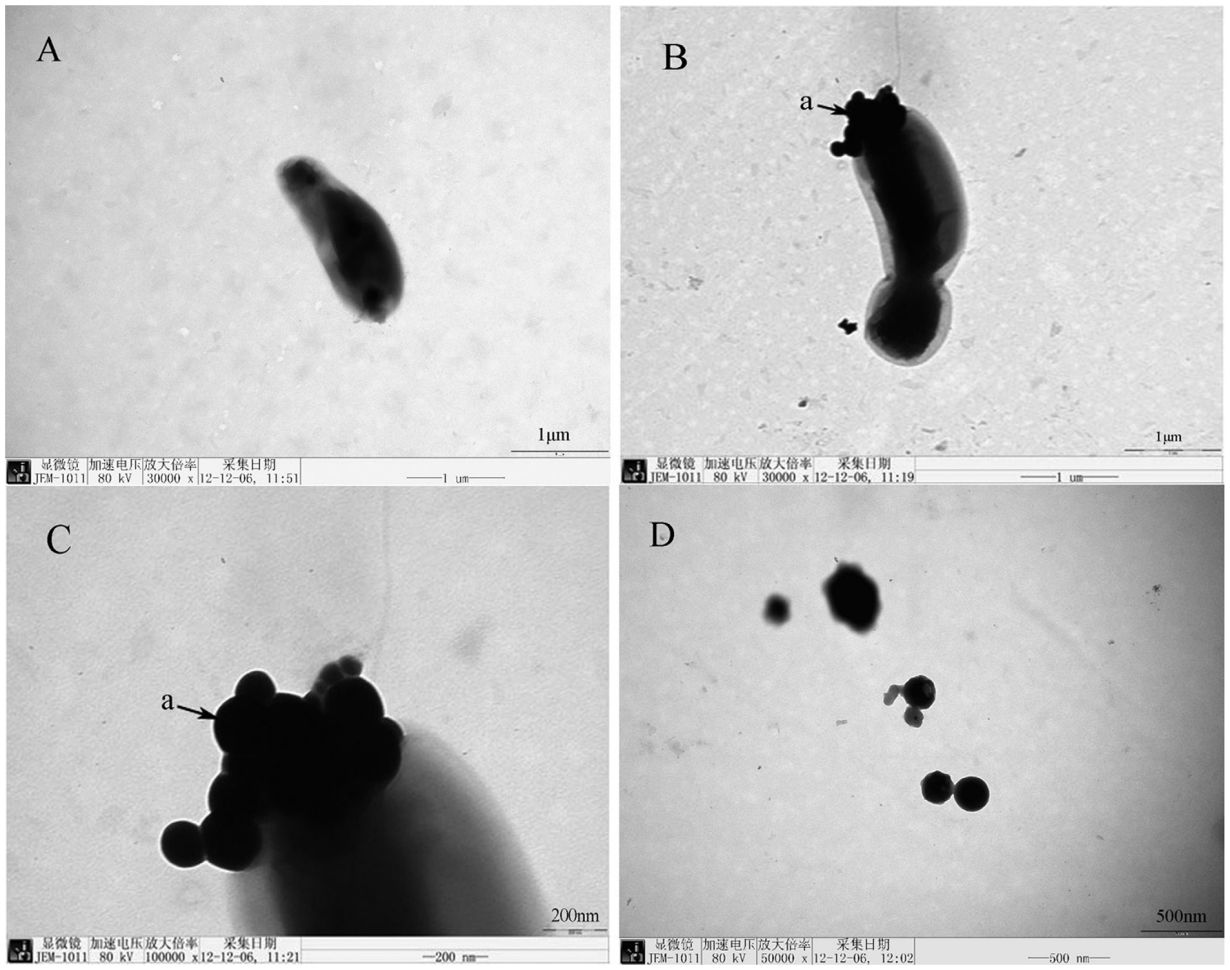
Transmission electron micrograph of *Rhodopseudomonas palustris* strain N. A, cells cultured without selenite; B–C, cells grown in the presence of selenite are showing electron-dense particles formed by the strain N; D, particles free in the medium, a: particles.

An electron micrograph of cells and selenium-containing particles obtained from the medium of a culture amended with 1.0 m mol/L SeO_3_^2−^ is shown in Fig. 3. In the control group, the strain surface was smooth (Fig. 3A). In the treated group, there were particles on the surface of stain (Fig. 3B), the shape of the particles was approximately spheric with a diameter of 80–200 nm (Fig. 3C). The particles free in the medium were observed also (Fig. 3D).

**Figure 4:**
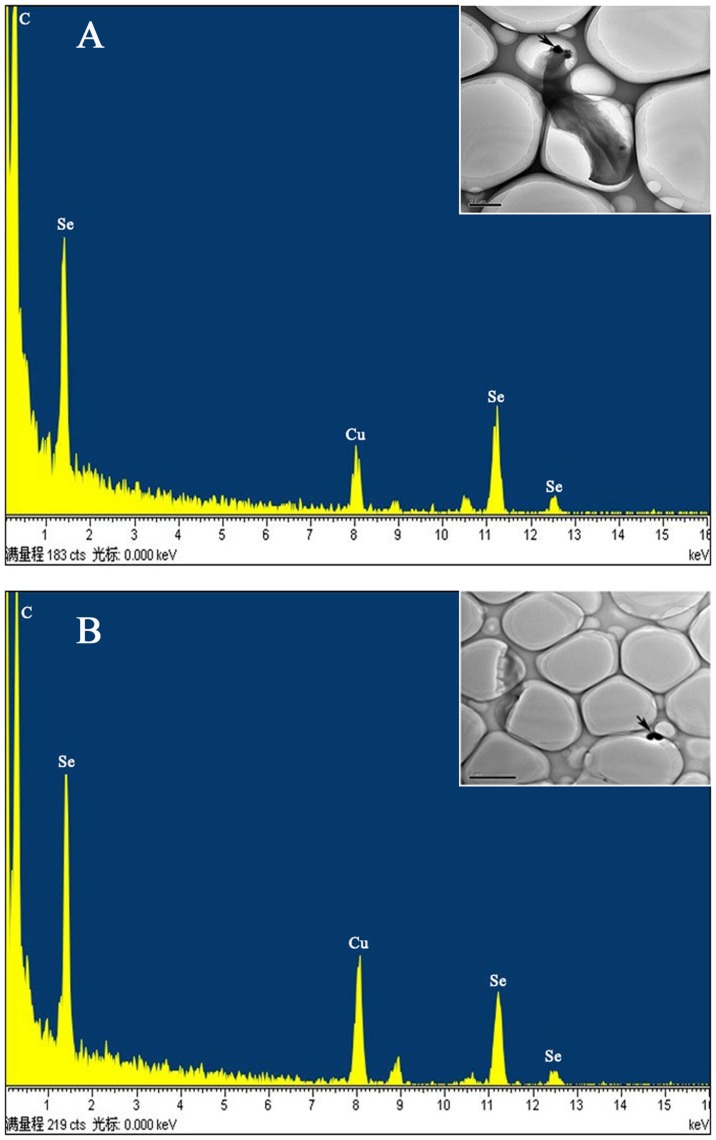
(A) Particles on the cell membrane. Energy levels (in kiloelectron volts) are indicated on the x axis. The emission lines for selenium are at 1.37 keV (peak SeLa), 11.22 keV (peak SeKa), and 12.49 keV (peak SeKb). (B) Particles in the culture medium. Energy levels (in kiloelectron volts) are indicated on the x axis. The emission lines for selenium are at 1.37 keV (peak SeLa), 11.22 keV (peak SeKa), and 12.49 keV (peak SeKb).

When preparations of obtained cells (Fig. 4A) and selenium-containing particles (Fig. 4B) were analyzed by using EDX analysis, the electrondense particles produced specific selenium absorption peaks at 1.37 keV (peak SeLa), 11.22 keV (peak SeKa), and 12.49 keV (peak SeKb). This result indicated that SeO_3_^2−^ was reduced to red elemental selenium by *Rhodopseudomonas palustris* strain N.

### X ray diffraction analysis

**Figure 5:**
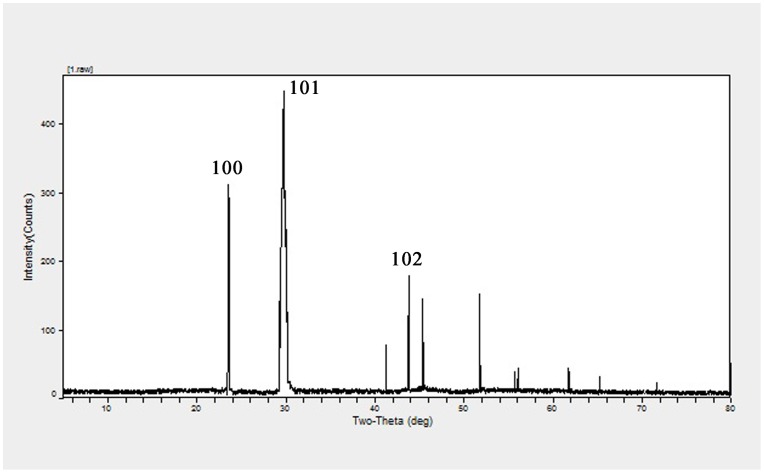
XRD pattern of selenium-containing particles formed by *Rhodopseudomonas palustris* strain N. The characteristic strong diffraction peak located at 29.777° is ascribed to the (101) facets of the face-centered cubic elemental Se^0^ structure.

The XRD pattern obtained for the extracellular red elemental selenium with three intense peaks in the whole spectrum of 2θ values ranging from 5 to 80 is shown in Fig. 5. The diffractions at 23.599°, 29.777° and 43.778° can be indexed to the (100), (101) and (102) planes of the face-centered cubic (fcc) red elemental selenium, respectively. The full-width-at-half-maximum (FWHM) values measured for 101 planes of reflection were used to calculate the size of the particles. The calculated average size of the red elemental selenium particles was determined to be 165 nm.

### Resistance to SeO_3_^2−^ Stress

**Figure 6:**
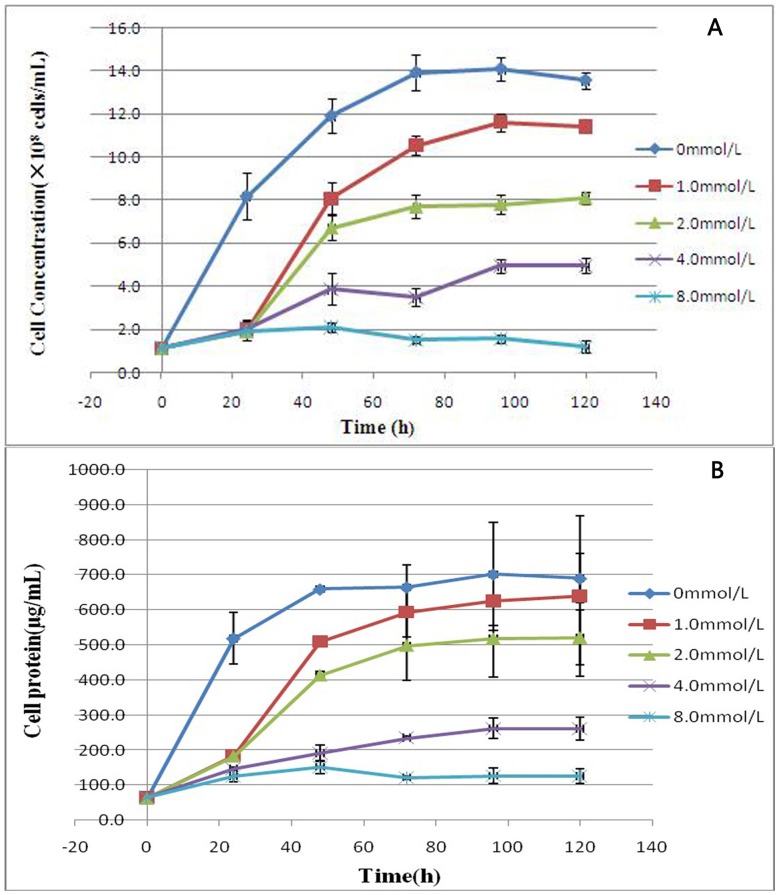
Time course of anaerobic phototrophic growth of *Rhodopseudomonas palustris* strain N in the presence of different selenite concentrations. (A) Cell concentration of the strain. (B) Protein content of the strain. Symbols: ⧫, control cells (no selenite); ▪, 1.0 m mol/L; ▴, 2.0 m mol/L; ×, 4.0 m mol/L; *, 8.0 m mol/L. Selenite was added at time zero. Each curve shows means based on the results of three experiments.

**Figure 7:**
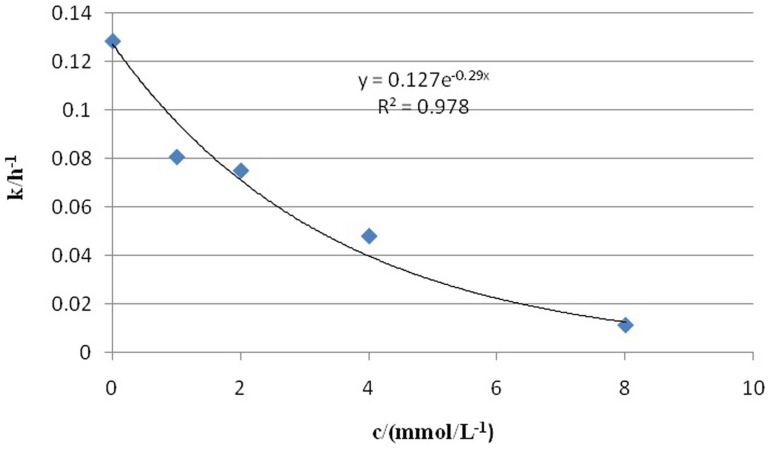
Influence of sodium selenite on the average growth rate of cells.

The results in Fig. 6 showed the SeO_3_^2−^ tolerance of *Rhodopseudomonas palustris* strain N at increasing concentrations of Na_2_SeO_3_. The cells were viable in the medium containing SeO_3_^2−^ and reached the end of log growth at 72 h after cultured. Growth kinetics data obtained at different SeO_3_^2−^ concentrations (1.0 to 8.0 m mol/L) showed that increasing the SeO_3_^2−^ concentration reduced the maximum attainable cell concentration (Fig. 6A) and protein content (Fig. 6B). The change trends of cell concentration were similar to the protein content of strain. The inhibition of the growth of bacteria increased with the increasing of SeO_3_^2−^ concentration in the medium. After 120 h, the presence of SeO_3_^2−^ up to 1 m mol/L resulted in 7.3% growth inhibition of the protein content of the control, whereas 24.5% (p<0.05), 62.1% (p<0.05) and 81.7% (p<0.05) growth inhibition was observed at 2.0, 4.0 and 8.0 m mol/L SeO_3_^2−^ concentrations, respectively. Based on the extent of growth inhibition (Fig. 6B), the effective concentration (EC_50_) of SeO_3_^2−^ was determined to be 2.4 m mol/L (k = 0.127exp(-0.29c), R^2^ = 0.978) (Fig. 7).

**Figure 8:**
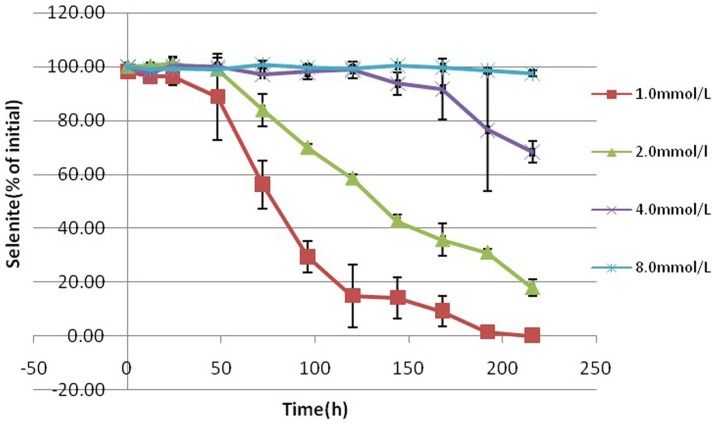
Time course of selenite reduction by *Rhodopseudomonas palustris* strain N during anaerobic phototrophic growth in the presence of different selenite concentrations. Symbols: ▪, 1.0 m mol/L; ▴, 2.0 m mol/L; ×, 4.0 m mol/L; *, 8.0 m mol/L. Selenite was added at time zero. Each curve shows means based on the results of three experiments.

The decrease in the SeO_3_^2−^ concentration during growth is shown in Fig. 8. This decrease was steep and complete at SeO_3_^2−^ concentrations of 1.0 m mol/L. In the culture amended with 2.0 m mol/L SeO_3_^2−^ reduction showed a similar slope as the slope in culture amended with 1.0 m mol/L SeO_3_^2−^, while the reduction kinetics of cultures containing 4.0 and 8.0 m mol/L SeO_3_^2−^ were different from them. After 9 d, the presence of SeO_3_^2−^ up to 1.0 m mol/L resulted in 99.9% reduction of SeO_3_^2−^, whereas 82.0% (p<0.05), 31.7% (p<0.05) and 2.4% (p<0.05) reduction of SeO_3_^2−^ was observed at 2.0, 4.0 and 8.0 m mol/L SeO_3_^2−^ concentrations, respectively. Furthermore, the start of the reactions was further delayed with increasing concentrations of SeO_3_^2−^.

### The location of elemental red selenium formation in grown cells

**Figure 9:**
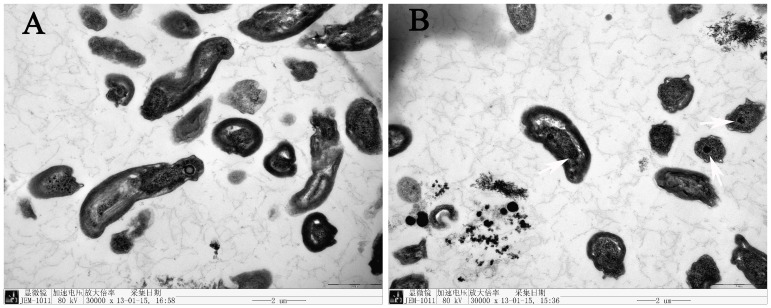
Transmission electron micrograph of the cells cultured without selenite (A) and exposed to 1.0 m mol/L selenite (B). Arrows indicate the paticles.

Under transmission electric microscope, high-electron-density particles having a regular geometrical shape were present in the cytoplasm of grown cells with SeO_3_^2−^ (Fig. 9). In addition, SeO_3_^2−^ was added to cell-free medium obtained from a 1.0 m mol/L SeO_3_^2−^ culture in which reduction was complete. The color of the solution did not changed at 30°C in the presence of incandescent light (1500 Lux) for 120 h. These results indicated that red elemental selenium formed in the cytoplasm of *Rhodopseudomonas palustris* strain N cells grown in the presence of SeO_3_^2−^.

## Discussion

Se^0^ is well known for its photoelectric, semiconductor, free-radical scavenging, anti-oxidative and anti-cancer properties [44]. The production of Se^0^ can be achieved through chemical and biological methods. Chemical detoxification of metals is proven to be very expensive and often results in secondary effects on the environment. The synthesis of Se^0^ using microorganisms has been suggested as a possible green method. In this study, *Rhodopseudomonas palustris* strain N was cultured in a medium containing SeO_3_^2−^ and particles obtained from the medium were proven to be Se^0^ by EDX and XRD analysis. The maximum concentration of SeO_3_^2−^ culture in which was complete of this strain was more than 1.0 m mol, and it was similar to *Bacillus megaterium* (≥0.25 m mol) [22] and *Rhodospirillum rubrum* (≥1.0 m mol) [31]. This study provided a new bacterial stain resource for the reduction of SeO_3_^2−^ to Se^0^.

In recent years, several different bacteria have been reported for the biological synthesis of Se^0^, such as *Thauera selenatis* [45] and *Rhizobium selenitireducens* strain B1 [23], [46], [47], *Escherichia coli* [48], *Clostridium pasteurianum* [49] and *Bacillus selenitireducens* [50]. In our study, *Rhodopseudomonas palustris* strain N also showed this ability. Moreover, the present *Rhodopseudomonas palustris* strain N belongs to the photosynthetic bacteria. The photosynthetic bacteria have more digestible bacterial cell wall, and are rich in protein, carotenoids, biological cofactors, and vitamins [51]. They were already shown to be suitable amendments to health foods for humans and animals. Therefore, reduction of SeO_3_^2−^ to Se^0^ by *Rhodopseudomonas palustris* strain N provides a potential application to artificially enrich food with selenium for human health.

Microorganisms have been shown to be particularly resistant to SeO_3_^2−^ [31], [32]. This resistance is attributed to the capacity of the organisms to reduce Se oxyanions to their elemental ground state. *Rhodopseudomonas palustris* strain N was resistant to the toxic effects of SeO_3_^2−^ and the minimum inhibitory concentration (the lowest amount of an antimicrobial agent that will completely inhibit the growth of an organism) for SeO_3_^2−^ for this organism exceeds 8 m mol/L. It was equal or greater than the MIC of other strains. For example, the MIC of *Bacillus subtilis* was 1.0 m mol/L [52], the MIC of *E. coli* W1485 was 5. 8 m mol /L [53], the MIC of *Bradyrhizobium japonicum* was 6.0–12.0 m mol/L [54], and the MIC of *Rhodobacter spheroids* was 2.9–4.6 m mol/L [55].

**Figure 10:**
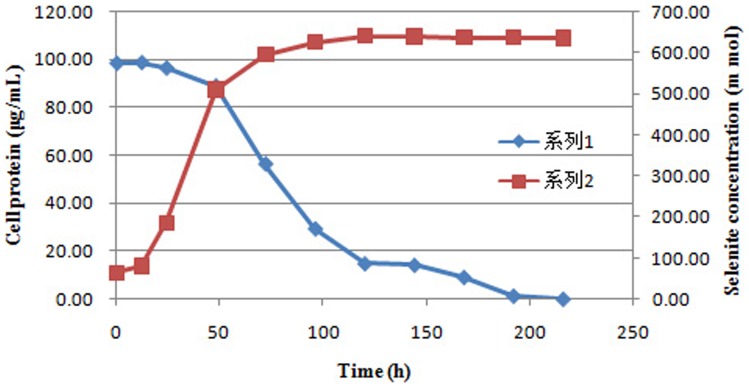
Electron micrograph of selenium-containing particles obtained after bacterial reduction in a culture amended with 1.0 m mol/L selenite.

Both electron micrographs showing intact cells after SeO_3_^2−^ reduction (Fig. 3B) and growth kinetics showing that the cell concentration and protein concentration in the stationary phase is rather constant (Fig. 6) suggest that cells were not severely damaged in the presence of SeO_3_^2−^ but rather viable. On the other hand, large amounts of selenium-containing particles present in the culture medium after SeO_3_^2−^ reduction (Fig. 10), indicating that *Rhodopseudomonas palustris* strain N is able to efficiently transport elemental selenium out of the cell. For collection of elemental selenium, this metabolism method was easier than others [56].

**Figure 11:**
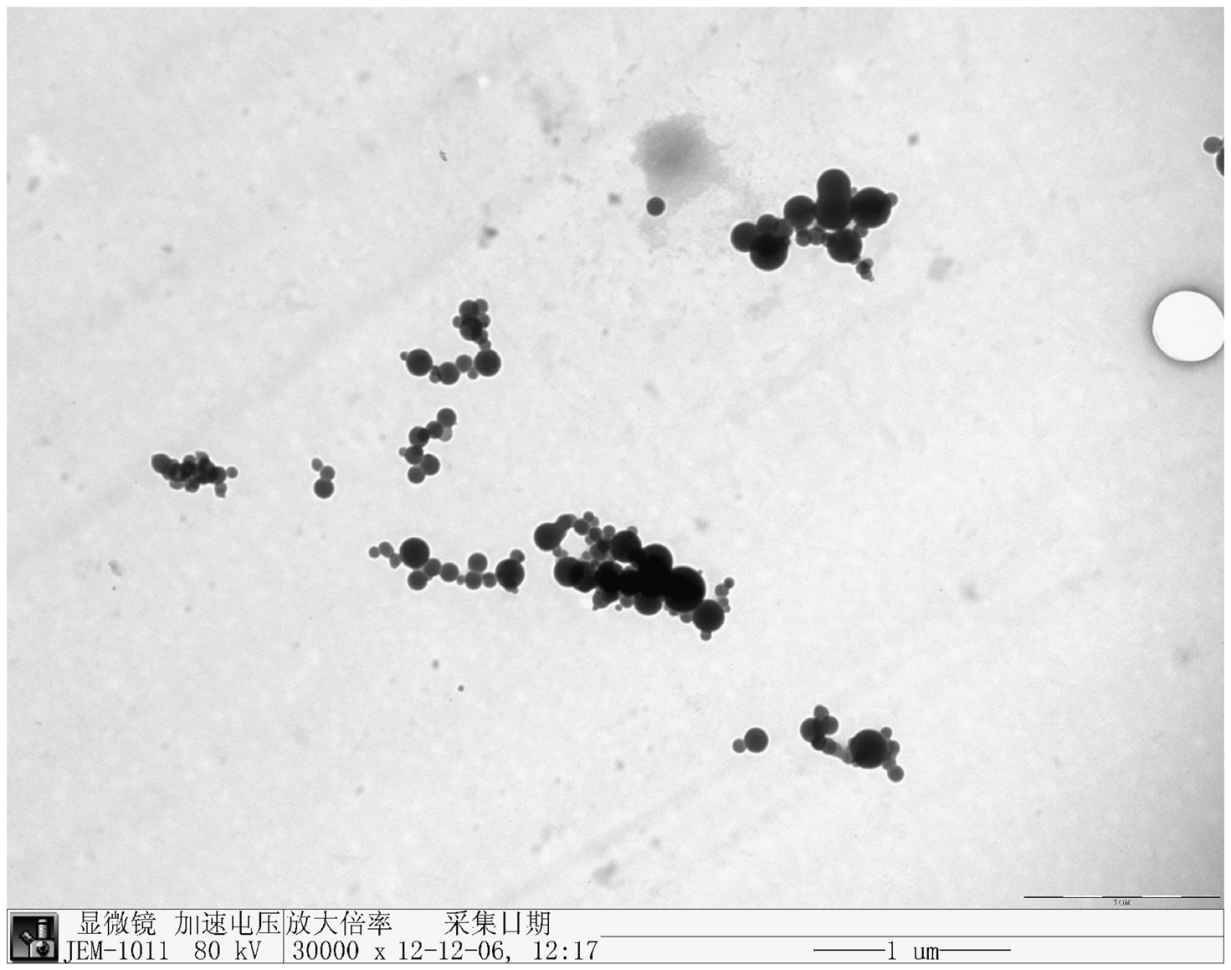
Time course of growth and selenite reduction by *Rhodopseudomonas palustris* strain N. Symbols: ▪, cell protein; ⧫, selenite concentration.

In our study, SeO_3_^2−^ reduction was closely related to the growth kinetics of cultures. It occurred at the end of the exponential phase and the stationary phase of bacterial growth (Fig. 11). This finding is similar to the results of Kessi et al. [31], who reported that the production of volatile selenium compounds occurred during the stationary phases. It means that the reduction is independent of strain growth. Moreover, this result suggests that reduction reaction is controlled by the stationary phase of the cultures.

In conclusion, we found that *Rhodopseudomonas palustris* strain N was capable of converting SeO_3_^2−^ to Se^0^. And this study provided a new microorganism resource and a potentially economically valuable ‘green’ synthesis route towards the synthesis of red elemental selenium, therefore contributing to the application of selenium for human health.
